# Feedback Inhibition Might Dominate the Accumulation Pattern of BR in the New Shoots of Tea Plants (*Camellia sinensis*)

**DOI:** 10.3389/fgene.2021.809608

**Published:** 2022-02-22

**Authors:** Hanghang Zhang, Dong Yang, Peiqiang Wang, Xinfu Zhang, Zhaotang Ding, Lei Zhao

**Affiliations:** College of Horticulture, Qingdao Agricultural University, Qingdao, China

**Keywords:** BR-accumulation pattern, BR biosynthetic pathway, feedback inhibition, new shoots of tea plants, negative correlation module

## Abstract

Brassinosteroid (BR), a kind of polyhydroxylated steroid hormone, plays an important role in physiological and biochemical processes in plants. Studies were mainly focused on BR signaling and its exogenous spraying to help enhance crop yields. Few research studies are centered on the accumulation pattern of BR and its mechanism. Yet, it is crucial to unlock the mystery of the function of BR and its cross action with other hormones. Tea (*Camellia sinensis* (L.) O. Kuntze) is one of the important economic crops in some countries, and new shoots are the raw materials for the preparation of various tea products. Different concentrations of exogenous BR were reported to have different effects on growth and development. New shoots of tea plants can thus be considered a valuable research object to study the accumulation pattern of BR. In this study, the quantity of five BR components (brassinolide, 28-norbrassinolide, 28-homobrassinolide, castasterone, and 28-norcastasterone) in different tissues of tea plants, including buds (Bud), different maturity of leaves (L1, L2), and stems (S1, S2) were determined by UPLC-MS/MS. A total of 15 cDNA libraries of the same tissue with three repetitions for each were constructed and sequenced. The BR-accumulation pattern and gene expression pattern were combined together for weighted gene co-expression network analysis (WGCNA). BR-accumulation-relative genes were then screened using two methods, based on the K.in value and BR biosynthetic pathway (ko00905), respectively. The result showed that photosynthesis-related genes and CYP450 family genes were actively involved and might play important roles in BR accumulation and/or its accumulation pattern. First and foremost, feedback inhibition was more likely to dominate the accumulation pattern of BR in the new shoots of tea plants. Moreover, three conserved miRNAs with their target transcriptional factors and target mRNAs had been figured out from negative correlation modules that might be strongly linked to the BR-accumulation pattern. Our study provided an experimental basis for the role of BR in tea plants. The excavation of genes related to the accumulation pattern of BR provided the possibility of cross-action studies on the regulation of BR biosynthesis and the study between BR and other hormones.

## Introduction

**FIGURE 1:**
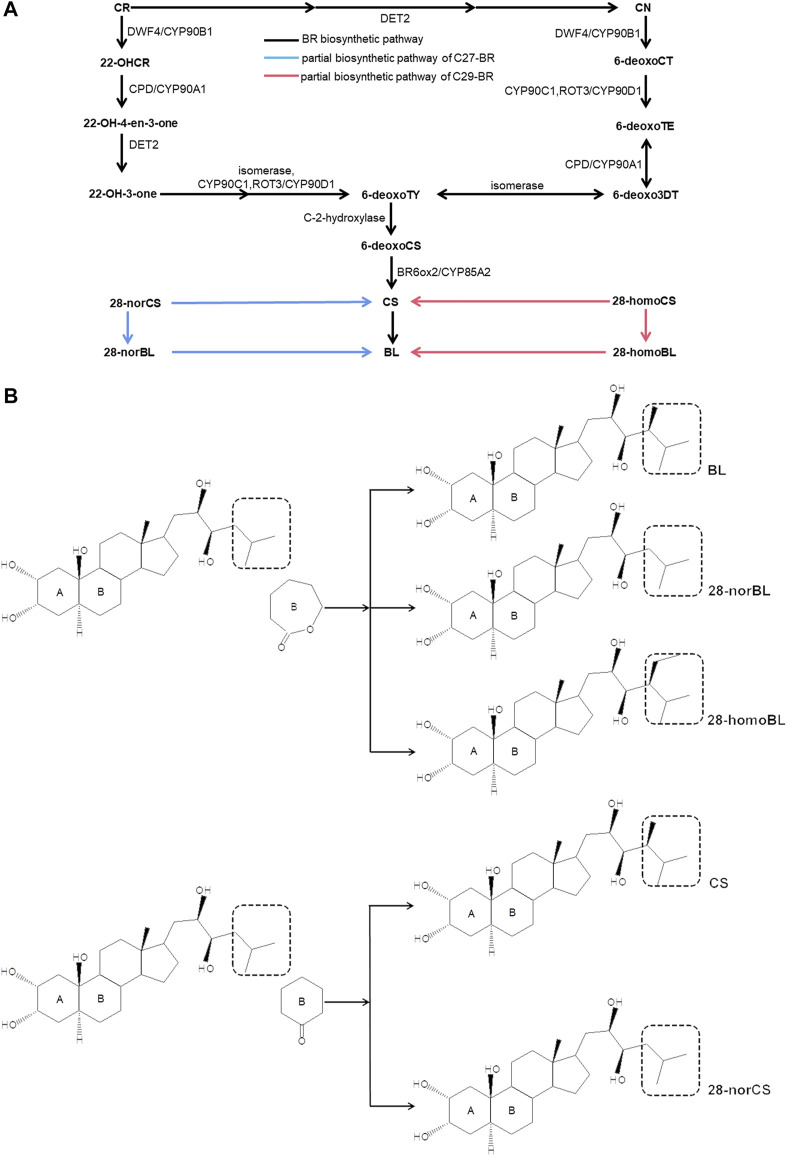
BR biosynthetic pathway. **(A)** BR biosynthetic pathway. **(B)** Structure of five BR (CS, 28-norCS, BL, 28-norBL, 28-homoBL). Campesterol (CR); campestanol (CN); 6-deoxocathasterone (6-deoxoCT); 6-deoxoteasterone (6-deoxoTE); 3-dehydro-6-deoxoteasterone (6-deoxo-3-DT); 6-deoxotyphasterol (6-deoxoTY); (22S)-22-hydroxycampesterol (22-OHCR); (22S,24R)-22-hydroxyergost-4-en-3-one (22-OH-4-en-3-one); castasterone (CS); brassinolide (BL); 28-norcastasterone (28-norCS); 28-homocastasterone (28-homoCS); 28-norbrassinolide (28-norBL); and 28-homobrassinolide (28-homoBL). The black box of a dashed line in the left could be replaced by the one in the right within the same brace in **(B)**.

Brassinosteroid (BR), known as the sixth natural phytohormone, is a polyhydroxylated steroid hormone (Liu L. et al., 2021). BR promotes cell elongation, cell division (Que et al., 2018; Oh et al., 2020; Conway et al., 2021), seed germination (Zhong et al., 2021), and fruit ripening (He et al., 2018) during growth and development, and it also plays an important regulatory role in plants under stress (Farooq et al., 2009; Procházka et al., 2016; Soares et al., 2020). A proven strategy to know how BR works is to figure out the biosynthesis pathway of BR in plants. At present, there are three main forms of BR in plants: C_27_-BR, C_28_-BR, and C_29_-BR (Bajguz et al., 2020). Many reactions, catalytic enzymes and biosynthetic genes have been excavated through the C_28_-BR biosynthetic pathway (Figure 1A), and it has been well understood. The C_28_-BR biosynthesis pathway starts from campesterol (CR), a biosynthetic substrate, and can be carried out through two parallel pathways, which are campestanol (CN)-dependent and CN-independent pathways, respectively. In the CN-dependent pathway, first, CR is catalyzed to form CN by enzymes such as DET2, and then CN → 6-deoxocathasterone (6-deoxoCT) →6-deoxoteasterone (6-deoxoTE) →3-dehydro-6-deoxoteasterone (6-deoxo-3-DT) → 6-deoxotyphasterol (6-deoxoTY), which are catalyzed by CYP90C1, ROT3/CYP90D1, CPD/CYP90A1, and isomerase, respectively. In the CN-independent pathway, CR directly forms 6-deoxoTY through a 5-step catalytic reaction as follows: CR→(22S)-22-hydroxycampesterol (22-OHCR)→(22S,24R)-22-hydroxyergost-4-en-3-one (22-OH-4-en-3-one)→(22S,24R)-22-hydroxy-5-ergostan-3-one (22-OH-3-one)→6-deoxoTY, and this process is catalyzed by DWF4/CYP90B1, CPD/CYP90A1, DET2, isomerase, CYP90C1, and ROT3/CYP90D1. The two pathways converge at 6deoxoTY, which is hydroxylated to 6-deoxocastasterone (6-deoxoCS). Then, 6-deoxoCS is catalyzed to form castasterone (CS). This step is catalyzed by BR6ox1*/*CYP85A1 and BR6ox2*/*CYP85A2. Finally, CS is catalyzed to BL by BR6ox2/CYP85A2. In addition, CR is finally transformed into 28-norbrassinolide (28-norBL) in the C_27_-BR pathway, and b-sitosterol is finally transformed into 28-homobrassinolide (28-homoBL) in the C_29_-BR pathway. 28-norcastasterone (28-norCS) and 28-homocastasterone (28-homoCS) in the two pathways are also the direct substrates of CS (Figure 1B) (Fujioka et al., 2000; Fujioka et al., 2002; Fujioka and Yokota, 2003; Kim et al., 2005; Fujita et al., 2010; Toshiyuki et al., 2012; Yuan et al., 2015; Toshiyuki and Ohnishi, 2018).

Although the BR biosynthetic pathway is relatively well understood, a central problem that cannot be ignored is that the phenomenon by which it accumulates in plants, and the reasons for its accumulation are not well understood. Recently, studies of BR were mainly focused on the signal transduction, the function of relative transcription factors (TFs), and enhancing crop yields by spraying exogenous BR (Divi and Krishna, 2009; Liu D. et al., 2021; Liu H. et al., 2021; Wang et al., 2021). It is reported that different concentrations of exogenous BR have different effects on plants, and gene expressions, and enzyme activities in the biosynthetic pathway of BR were inhibited when the accumulation of BR in plants became high (Ha et al., 2018). That is, the accumulation of BR may play a subtle and important coordinating role in maintaining BR and/or balancing the level of BR and other hormones. Thus, it is necessary and important to understand the accumulation pattern of BR and its mechanism. The tea plant (*Camellia sinensis* (L.) O. Kuntze) has become one of the important economic crops in many countries, such as China, India, and Japan (Liu Z. et al., 2021). In addition, studies reported the application of exogenous BR could improve the tea quality and yield (Li et al., 2016; Li et al., 2017; Li et al., 2018). They also found that different concentrations of BR have different effects on tea plants. Meanwhile, new shoots of tea plants are the major raw materials for the processing of various tea products (Zhang Z. et al., 2021). New shoots of tea plants can thus be considered a valuable research object to study the accumulation of BR and their accumulation patterns.

**FIGURE 2:**
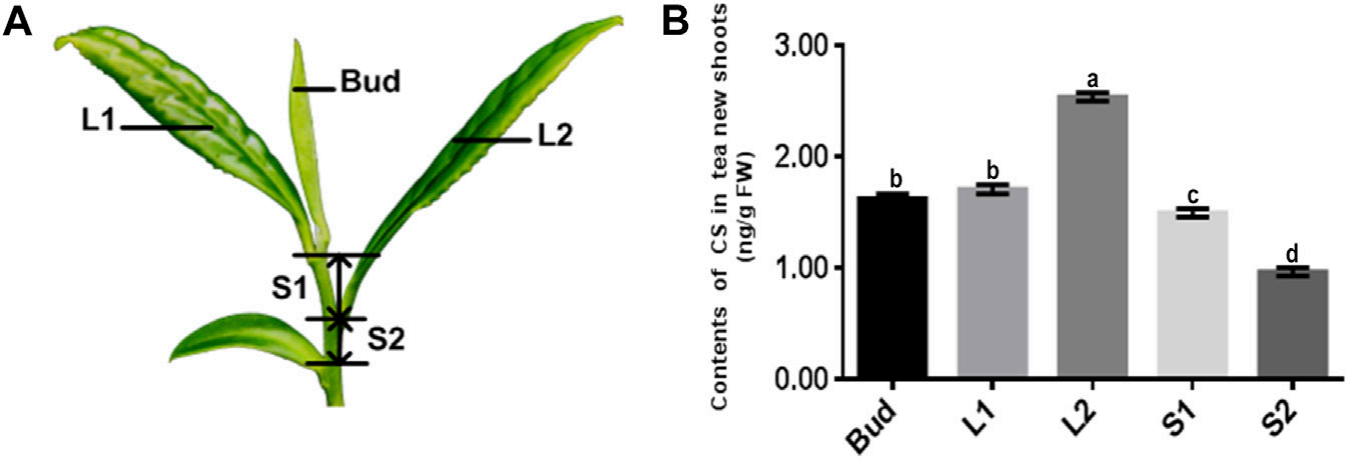
The content of CS in different tissues of new shoots in tea plants. **(A)** Bud, leaf, and stem samples were collected from top to bottom, named bud, L1, L2, S1, and S2. **(B)** Content of CS in five tissues of tea tree’s new shoots. Data values are the mean ± SD of three independent biological samples. Different letters above the bars indicate significantly different values (*p* < 0.05).

In this study, five main components of BR, brassinolide (BL), 28-norbrassinolide (28-norBL), 28-homobrassinolide (28-homoBL), castasterone (CS), and 28-norcastasterone (28-norCS) (Figure 1B) in different tissues of new shoots in tea plants (Figure 2A) were determined by using ultra-performance liquid chromatography–tandem mass spectrometry (UPLC-MS/MS). The RNA-seq data of five tissues from the new shoots of tea plants and the accumulation mode of BR were banded together to mine potential genes involved in BR biosynthesis. Then, the connectivity of genes involved in the accumulation mode of BR was sorted by two different methods, which were from the perspective of the K.in value and BR pathway (ko00905), respectively. The genes related to the accumulation pattern of BR involved in metabolic pathways were then enriched by the analysis of Kyoto Encyclopedia of Genes and Genomes (KEGG). The main BR-accumulation pattern and connections between the relative genes and their potential roles in effecting the accumulation pattern of BR were discussed. Our study on the contents of BR-related substances and the accumulation pattern will provide an experimental basis for the role of BR in tea plants. Moreover, the excavation of genes related to the accumulation pattern of BR will provide the possibility of cross-action studies on the regulation of BR biosynthesis and the study between BR and other hormones.

## Methods and Materials

### Plant Materials

Four-year-old Pingyang Tezaocha (PYTZ) (*Camellia sinensis* (L.) O. Kuntze) tea plants were grown in an experimental research garden at Qingdao Agricultural University (latitude 35°N, longitude 119°E, Qingdao city, China) under natural conditions. Samples of five different tissues were collected from top to bottom: bud (Bud), the first leaf below the bud (L1), the second leaf with higher maturity below the bud (L2), the stem between the first leaf and the second leaf (S1), and the more mature stem between the second leaf and the fish leaf (S2) were collected from the top to bottom in order (Figure 2A). The standards of the samples could be referred to the previous published experimental method (Zhao et al., 2019). For each operation three repetitions were needed, with each repetition conducted on at least three tea seedlings. These samples were immediately frozen in liquid nitrogen after collection and stored in a −80°C refrigerator for extraction and quantification of BR, RNA isolation for sequencing, and qRT-PCR as described below.

### Extraction and Quantification of BR

Plant tissue (1 g) (fresh samples of Bud, L1, L2, S1 and S2 mentioned above) was grounded to powder form with a grinding machine, and then, transferred into a 10-ml centrifuge tube. Stable isotope-labeled BR [^2^H3] BL (1 ng/g) and [^2^H3] CS (1 ng/g) were added into the mixture followed by extraction with acetonitrile (5 ml) for 12 h in a refrigerator at −18°C. The acetonitrile exacted sample was centrifuged at 10,000 g under 4°C for 20 min. Then, the extracted sample was blown dry with nitrogen and re-dissolved with acetonitrile. All the obtained solutions were combined, and graphitized carbon black, C_l8_-bonded silica, and ethylenediamine-N-propyl-bonded silica were added, vortexed, mixed for 5 min, and centrifuged to collect the super sample effluent. The oleoresin sterols adsorbed on the material were then desorbed with methanol (2 ml), the upper sample effluent and desorption solution were combined, and the solvent was evaporated. The sample residue was dissolved with a buffer solution (20 mM ammonium formate acetonitrile/water, 1/4, V/V, pH9, 5 ml). The five compound standards of BR, BL, 28-norBL, 28-homoBL, CS, and 28-norCS and stable isotope-labeled standards [^2^H3] brassinolide and [^2^H3] castasterone were purchased from Olchemim Ltd (Olomouc, Czech Republic). All solutions were extracted with pre-activated phenylboronic acid magnetic condensation (20 mg) and were centrifuged, and the supernatant was discarded. After washing with acetonitrile containing 0.5% ammonia, the extracted BR was desorbed with a water/acetonitrile solution (1/9, V/V) containing 3% H_2_O_2_, and after blowing the solvent dry, the volume was fixed to 100 μl, of which 80 μl was used for subsequent UPLC-MS/MS analysis (Ding et al., 2013).

### Library Construction, Sequencing, and Data Analysis

Total RNA for each sample (Bud, L1, L2, S1, and S2 mentioned above) was isolated using RNAiso-mate and a plant tissue kit (Takara, Tokyo, Japan). The quality, purity, concentration, and integrity of the RNA were accordingly checked. RNA samples with a 260/280 ratio around 1.8, 260/230 ratio around 2.0, and RNA integrity number more than 8.0 were used for sequencing and quantitative analysis. For mRNA library construction, total RNA were first enriched by using Oligo (dT) beads, fragmented into short fragments using a fragmentation buffer, and then reverse transcribed into cDNA with random primers. Then, the enriched mRNA and second-strand cDNA were synthesized by using DNA polymerase I, RNase H, dNTP, and a buffer. Then, the cDNA fragments were purified with a QiaQuick PCR extraction kit, end repaired, poly (A) added, and ligated to Illumina sequencing adapters. The ligation products were size selected by agarose gel electrophoresis, PCR amplified, and sequenced on an Illumina HiSeq™ 3000 sequencer using PE150 method by RiboBio Co., Ltd (Guangzhou, China). Raw reads containing adapters, low quality bases, and short reads that could be mapped to the ribosome RNA were removed before assembly and further analysis in the transcriptome. Then, the clean reads of each sample were mapped to the reference genome (NCBI Sequence Read Archive Database under accession number PRJNA381277) by Tophat2 (Trapnell et al., 2010). Gene abundances were quantified by using software RSEM (Bo et al., 2011). Data analysis was performed by Gene Denovo Biotechnology Co. (Guangzhou, China). The fragments per kilobase of transcript per million mapped reads (FPKM) were used to calculate gene expression among samples. For the significance analysis of differences, the two-tailed student’s test was used to estimate the significance of the difference between the pairwise comparisons. Using the SPSS statistical program, the one-way ANOVA was used to compare the statistical significance of the differences between the groups, and then the least significant difference post hoc test was performed when *p* < 0.05.

### Weighted Gene Co-Expression Network Analysis and Visualization of Gene Networks

WGCNA is a system biology method for hierarchical clustering and identification of co-expressed genes. The R software package (v1.47) of WGCNA was used to calculate the intra module connectivity and module correlation (MM) of each gene. Firstly, it was assumed that the gene network follows a scale-free distribution, and then the adjacency matrix was further transformed into a topological overlap matrix (TOM), so as to establish a hierarchical clustering tree (Langfelder and Horvath, 2008). The generated gene tree was cut and distinguished by dynamic tree cutting method (minimum module size = 50) to produce different modules. After the preliminary module division, the modules with similar expression patterns were combined according to the module similarity to form a dynamic tree. Subsequent analyses were carried out according to the combined module (Esposito et al., 2021). The KEGG pathway enrichment analysis was performed using the KEGG database (http://www.genome.jp/kegg/) (Minoru et al., 2008). After multiple test correction, the path with Q value ≤0.05 was defined as significantly enriched genes. The Q value was the correction of *p*-value after FDR (Benjamini and Hochberg, 1995). We screened the genes of interest in the positive and negative correlation modules separately, then screened the relationship pairs of miRNAs and TFs and their target mRNAs, and finally, constructed the co-expression network using Cytoscape version 3.7.2 (http://www.cytoscape.org/).

### Quantitative Real-Time Polymerase Chain Reaction Analysis

Total RNA was isolated from Bud, L1, L2, S1, and S2 by using an RNA extraction kit (Code No. 9108, Takara, Tokyo, Japan), and then converted to cDNA by using a PrimeScript^®^RT reagent kit according to the manufacturer’s protocol (Code No. RR047A, Takara, Tokyo, Japan). *GAPDH* was used a normalization reference. Gene specific primers of the twelve selected genes were designed by using primer5. All the primer sequences were also shown in Supplementary Table S3. The qRT-PCR was carried out on a CFX96 Real-Time PCR detection instrument (Bio-Rad, Inc., Hercules, CA, United States) with a SYBR Premix Ex Taq^TM^II Kit (Tli RNase H Plus) (Code No. RR820A, Takara, Tokyo, Japan). The reaction mixtures were studied under the following parameters: 95°C for 30 s, 30 cycles at 95°C for 5 s, 60°C for 30 s. The relative expression of genes were calculated by using the 2^ ^−∆∆Ct^ analysis method (Livak and Schmittgen, 2001). The qRT-PCR of each gene was performed with three technical replicates and biological replicates. The calculation method of significance analysis was the same as the above mentioned in 2.3.

## Results

### Low Contents of BR Biosynthetic Metabolites and Their Distribution in the New Shoots of Tea Plants

The contents of five main substances of BR biosynthetic metabolites (Supplementary Table S1), BL, 28-norBL, 28-homoBL, CS, and 28-norCS had been detected in the new shoots of tea plants via UPLC-MS/MS. Only CS and 28-CS could be quantitated, even so, their contents were quite low (Supplementary Table S1). Within that, 28-CS could not be checked out in sample S2. The accumulation pattern of CS in the five samples of tea plants at different developmental stages was thus to be considered the key point. By quantitative analysis, the results showed that the contents of CS in Bud, L1, L2, S1, and S2 were 1.63, 1.71, 2.54, 1.50, and 0.97 (ng/g, FW), respectively (with ±0.04 error interval). Through the data, the content of CS in mature leaves (L2) was more than that in tender leaves (L1), the content of CS in old stems (S2) was less than that in tender stems (S1), and the content of CS in bud (Bud) was more than that in stems (S1, S2) (Figures 2A, B).

### Expression Characters of Whole Genes in the New Shoots of Tea Plants

To figure out candidate genes that may affect the accumulation of BR metabolites in the new shoots of tea plants, the transcriptome of buds, leaves, and stems had been sequenced and analyzed. The qualities of fifteen libraries, including the total length of clean reads, the percentages of Q20 and Q30, percentage of GC content, and the ratio of mapping to reference genome, met the requirements of subsequent analyses and are shown in Supplementary Table S2. The pearson correlation analysis showed a high consistency among the replicates, suggesting that the transcriptome data deserves to be relied upon for subsequent studies (Supplementary Figure S1).

### Focalization of BR-Accumulation–Related Genes Based on the K.in Value

**FIGURE 3:**
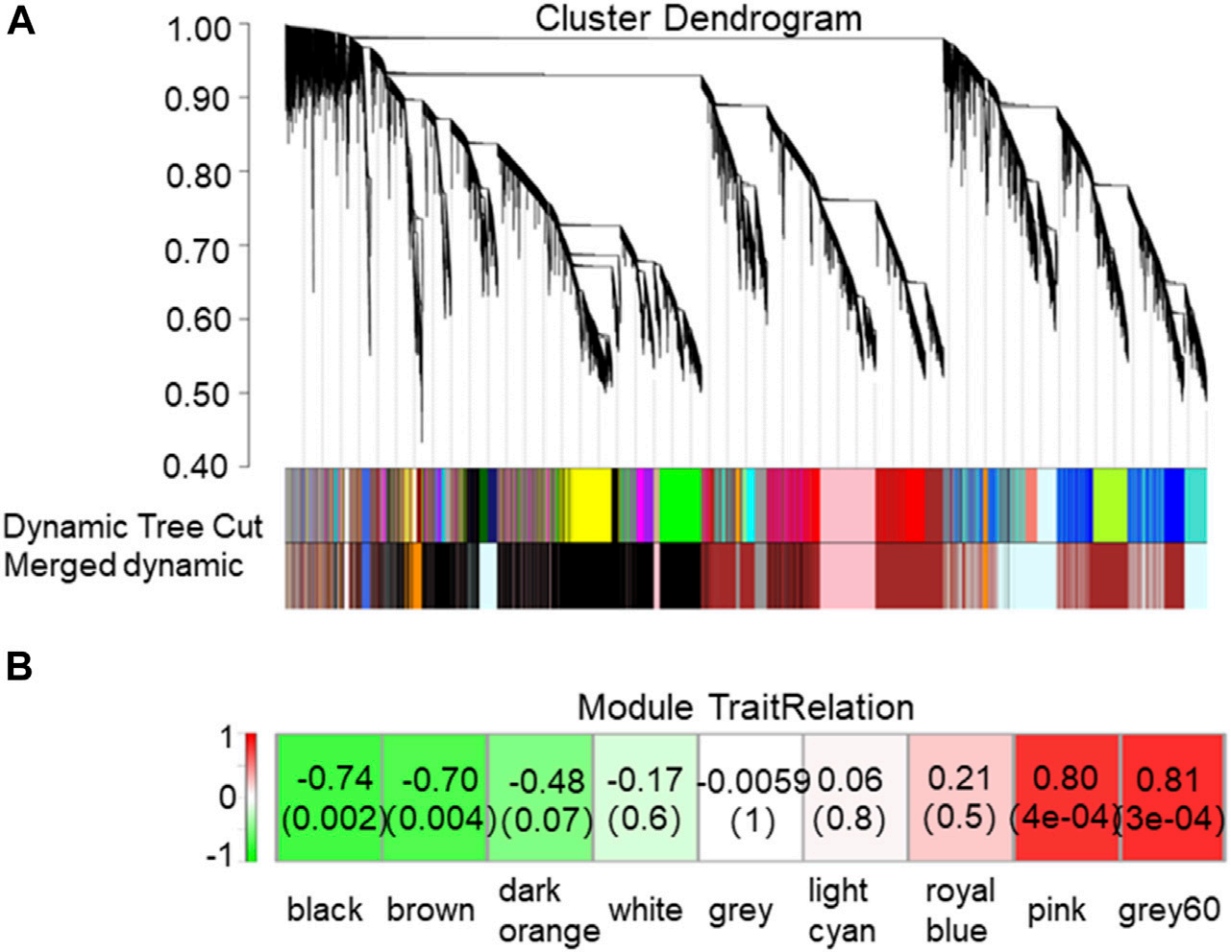
Network analysis dendrogram showing modules identified by weighted gene co-expression network analysis (WGCNA). **(A)** Nine modules, each associated with a leaf in the tree corresponds to an individual gene. **(B)** Association between modules and traits. The color of each module is the same as that in **(A)**. The left column represents the color scale for module/feature dependencies from -1 to 1. Each cell in the right row contains the corresponding number of correlations and *p*-values. The bottom line represents the module name corresponding to the cell above.

To explore the genes in tea plants that may be related to the accumulation of metabolites in the BR biosynthetic pathway, the correlation between the content of CS in the BR biosynthetic pathway and the expression of all genes from the transcriptome of new shoots in tea plants was analyzed by applying WGCNA. The results showed that according to the content of CS, all genes could be divided into nine modules (Figure 3), among which, “grey60” (r = 0.81, *p* = 3e-04) and “pink” (r = 0.8, *p* = 4e-04) were positive correlation modules, involving 600 and 2326 genes, respectively. Meanwhile, “black” (r = −0.74, *p* = 0.002) and “brown” (r = −0.7, *p* = 0.004) were negative correlation modules, involving 8605 and 8262 genes, respectively.

**FIGURE 4:**
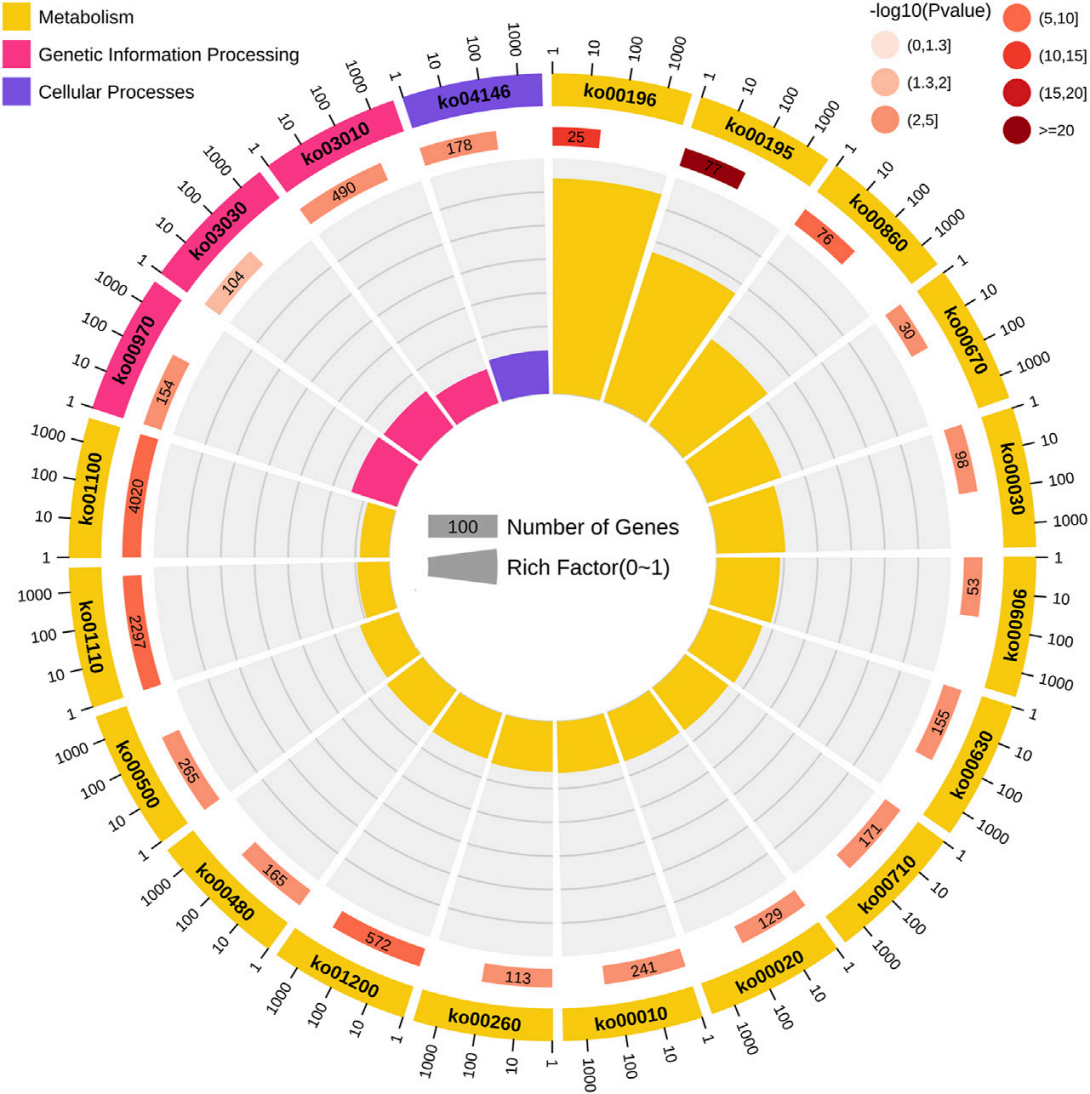
Kyoto Encyclopedia of Genes and Genomes (KEGG) pathway enrichment analysis of genes among the four modules with high correlation. From the outermost circle to the inner circle: the first circle represents the path of the top 20 enrichment, and the outside of the circle is the coordinate ruler of the number of genes. Different colors represent different classes. Yellow represents metabolism; pink represents genetic information processing; and purple represents cellular processes. The second circle represents the numbers and *p*-values of the pathways in the background genes. The more the background numbers of the genes, the longer the bar, and the smaller the *p*-values, the redder the color. The third circle represents the rich-factor value of each pathway (the number of genes in the pathway divided by all the numbers in the path).

The screening of the key genes (hub genes) in the positive and negative modules was according to the comprehensive consideration of the degree of module correlation and connectivity within the module (all .k within the “K.in value”). The higher the absolute value of the “K.in value”, the more active the function or core position of the gene might have/be. Based on this, the number of “grey60”, 600, was used as the base number and top 600 genes from each other three modules was selected according to the K.in value to perform the KEGG analysis (Figure 4). The result showed that in the metabolism pathway, genes were significantly enriched in three biosynthetic pathways: photosynthesis-antenna proteins (ko00196), photosynthesis (ko00195), and porphyrin and chlorophyII metabolisms (ko00860). To further find out what kinds of specific annotation of high K.in value genes in each module have, ten genes with the highest K.in value as hub genes in each of the two positively correlated modules (“grey60” and “pink”) was selected and analyzed (Table 1). Nine genes in the positive correlation modules were associated with chloroplasts and photosynthesis, including two in “grey60” and seven in “pink” was filtered. The same analysis was performed to the two negative modules of “black” and “brown”. It was worth noting that eight genes were also turned out to be associated with chloroplasts and photosynthesis, in which one belonged to “black” and seven belonged to “brown” (Table 1). Usually, chloroplast-related genes belonging to the photosystem has majority participation in the process of photosynthesis.

**TABLE 1:** List of top ten genes in each module with the K.in value.

Gene ID	Module	K.in value	Annotation	Correlation
XLOC_039279	grey60	239.39	Cold-inducible protein	+
CSA022417	grey60	236.6	Uncharacterized protein LOC109215671	+
CSA011542	grey60	236.08	Nitrate/nitrite transporter, partial	+
XLOC_049288	grey60	236.06	Transmembrane protein 64	+
CSA029944	grey60	231.68	Photosystem I reaction left subunit psak, chloroplastic	+
CSA001272	grey60	230.42	Catalase	+
CSA014792	grey60	230.31	DUF1230 family protein	+
CSA008965	grey60	229.03	Mannitol dehydrogenase isoform X2	+
CSA020118	grey60	228.26	Uncharacterized protein ycf36	+
CSA027463	grey60	228.14	Calcium sensing receptor, chloroplastic isoform X2	+
CSA002865	pink	1,033.47	30S ribosomal protein S20, chloroplastic	+
CSA006070	pink	1,032.26	Ribosomal protein L27, partial	+
CSA032812	pink	1,031.36	Ribosome-recycling factor, chloroplastic	+
CSA009530	pink	1,031.34	Peptidyl-prolyl cis-trans isomerase FKBP13, chloroplastic	+
CSA008991	pink	1,028.17	RNA polymerase sigma factor sigF, chloroplastic	+
CSA005377	pink	1,027.50	Peptidyl-prolyl cis-trans isomerase FKBP18, chloroplastic	+
CSA011770	pink	1,027.36	50S ribosomal protein L21, chloroplastic	+
CSA024492	pink	1,027.29	MFS_1 domain-containing protein	+
CSA033261	pink	1,026.24	2,3-bisphosphoglycerate-independent phosphoglycerate mutase	+
CSA026125	pink	1,026.12	50S ribosomal protein L6, chloroplastic-like	+
XLOC_050324	black	1,159.34	Patatin-like protein 2 isoform X2	−
CSA016896	black	1,158.50	ABC transporter B family member 1	−
XLOC_016866	black	1,154.62	Transcription factor PIF3 isoform X1	-
CSA019420	black	1,148.93	ABC transporter B family member 11	−
CSA025592	black	1,148.16	Reticulon-like protein B12 isoform X1	−
CSA009335	black	1,147.88	Cytochrome b561 and DOMON domain-containing protein At3g25290-like	−
CSA014717	black	1,146.41	Alpha-glucosidase	−
CSA019338	black	1,145.68	Auxin efflux facilitator isoform 1	−
CSA005938	black	1,145.31	COP1-interacting protein 7	−
CSA010064	black	1,144.99	Aldehyde dehydrogenase	−
CSA010862	brown	879.87	Chlorophyll a-b binding protein CP29.1, chloroplastic	−
CSA018950	brown	879.17	Glyceraldehyde-3-phosphate dehydrogenase B, chloroplastic	−
CSA016587	brown	878.46	Chlorophyll a-b binding protein 8, chloroplastic	−
CSA035815	brown	874.41	Cytochrome b6-f complex iron-sulfur subunit, chloroplastic	−
CSA027742	brown	872.59	Thioredoxin-like protein AAED1, chloroplastic isoform X2	−
CSA027395	brown	872.42	DUF1620 domain-containing protein/PQQ_2 domain-containing protein	−
CSA010617	brown	871.96	Serine hydroxymethyltransferase, mitochondrial	−
CSA007481	brown	871.84	Uncharacterized protein LOC100256501 isoform X3	−
CSA006075	brown	871.75	Photosystem I reaction left subunit XI, chloroplastic	−
CSA026004	brown	871.22	Photosystem II PsbQ, oxygen evolving complex	−

Interestingly, the K.in value of “pink” was much higher than that of “grey60” in the positive modules, and the K.in value of “black” was much higher than that of “brown” in the negative modules. In addition, the K.in value of negative modules was higher than that of positive modules. Thus to sum up for this result, genes in the negative modules were more likely to have more active function or were in the core position, and photosynthesis genes were intensively enriched in affecting the pattern of BR metabolites (Table 1).

### Focalization of BR-Accumulation–Related Genes Based on the BR Biosynthetic Pathway (ko00905)

The above part of the analysis was based on the annotation of genes with a high K.in value in the positive and negative modules. In this section, we selected the BR-accumulation–related genes again, first and foremost based on the BR biosynthetic pathway (ko00905) and then considered and combined with their K.in value and expression levels. The analysis began with ko00905 of the BR biosynthetic pathway, screened all the genes in the pathway, then ranked them based on the K.in value, and removed the genes with fairly low expression levels. Twenty-three genes in the BR biosynthetic pathway may contribute significantly to the accumulation mode of CS (Table 2). Among them, twenty-one genes belonged to the negative modules black and brown, and two genes belonged to the positive module “grey60”. Among the twenty-one genes in the negative modules, eight genes belonged to the “black”, and all were CYP450 family members, *CYP734A51*, *CYP734A1*, *CYP749A48*, and *CYP724B1-like*. The other thirteen genes belonged to the “brown”, in which ten genes belonged to the CYP450 family, *CYP90B1*, *CYP90A2*, *CYP90B1-like*, *CYP85A1*, *CYP749A22-like*, *CYP724B1 isoform X2*, and *CYP450*; three genes belonged to the BR biosynthetic pathway; two were *3-epi-6-deoxocathasterone 23-monooxygenase*; and the other one was coding bromo-adjacent homology (BAH) domain–containing protein. Both genes in the positive module belonged to “grey60”, one of which was *3-epi-6-deoxocathasterone 23-monooxygenase*, and the other gene was *brassinosteroid-6-oxidase*. The electron expressions of the twenty-three genes calculated by their fragments per kilobase of transcript per million mapped reads (FPKM) could be found in Supplementary Figure S2.

**TABLE 2:** List of genes with high expression and the K.in value in ko00905.

Gene ID	K.in value	Annotation	Module	Correlation
CSA019449	688.03	CYP734A51	black	−
XLOC_044752	611.12	CYP734A1	black	−
CSA013522	609.44	CYP734A1	black	−
CSA008763	233.44	CYP749A48	black	−
XLOC_050369	204.78	CYP749A48	black	−
CSA008764	161.37	CYP749A48	black	−
XLOC_037237	130.64	CYP749A48	black	−
XLOC_038759	103.99	CYP724B1-like	black	−
CSA034027	760.35	CYP90B1	brown	−
CSA017075	648.72	CYP90A2	brown	−
CSA017249	609.23	CYP90A2	brown	−
CSA008631	547.5	3-epi-6-deoxocathasterone 23-monooxygenase	brown	−
XLOC_017809	531.36	CYP90B1-like	brown	−
CSA011358	457.03	CYP85A1	brown	−
XLOC_027275	135.58	3-epi-6-deoxocathasterone 23-monooxygenase	brown	−
XLOC_044989	125.89	CYP749A22-like	brown	−
XLOC_036921	103.89	CYP724B1 isoform X2	brown	−
XLOC_030548	67.31	CYP749A22-like	brown	−
XLOC_039435	45.02	CYP749A22-like	brown	−
XLOC_038760	30.53	CYP450	brown	−
CSA019803	3.04	Bromo-adjacent homology (BAH) domain–containing protein	brown	−
CSA008632	109	3-epi-6-deoxocathasterone 23-monooxygenase	grey60	+
CSA035177	5.27	Brassinosteroid-6-oxidase	grey60	+

Thereby making a summary for this result, genes in the negative modules were likely to have a crucial effect, and the genes belonged to CYP450 family, may play a key role in affecting the BR-accumulation pattern. It was evident from these results that, regardless of which analysis methods were performed, the important genes affecting the accumulation pattern of BR metabolites and gene expression patterns were mainly distributed in the negative correlation modules.

### qRT-PCR Verification of Gene Expressions in Positive/Negative Modules

**FIGURE 5:**
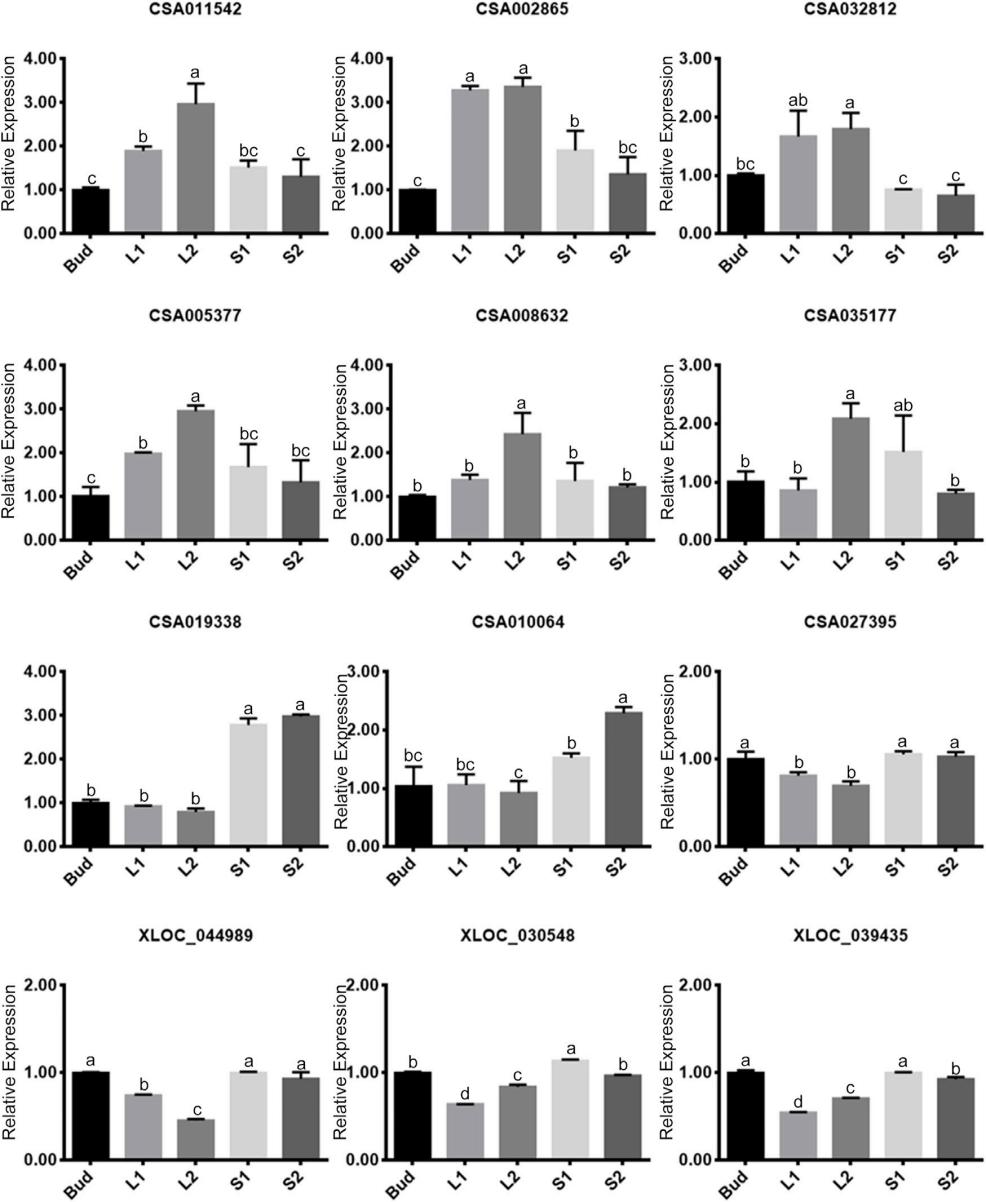
Relative expressions of twelve selected genes by quantitative real-time polymerase chain reaction (qRT-PCR). The former six genes belong to positive modules, and the later six genes belong to negative modules. Data values are the mean ± SD of three independent biological samples. Bars labeled with different lower case letters mean significant difference (*p*-value less than 0.05), while with the same ones mean insignificance (*p*-value higher than 0.05).

To verify the expression of genes that were screened in the above four modules, twelve genes were selected representatively for qRT-PCR, of which six belonged to the positive modules and six belonged to the negative modules (Figure 5). As it was shown, the expression levels of the twelve genes showed significant differences. In the positive correlation modules, the expression of each gene (the former six genes) in L2 was higher than that in any other tissues. Five of the selected genes showed a trend of rising in Bud–L1–L2, and then decreasing in L2–S1–S2. In the negative modules, the expression of each gene (the later six genes) showed a different trend compared with that in the positive modules. We had further confirmed their consistency in the transcriptome based on FPKM.

## Discussion

### Photosynthetic Genes Played an Active Role in the BR-Accumulation Pattern in the New Shoots of Tea Plants

So far, many studies had reported that phytohormones of BR can regulate plant growth and development and respond to stress through photosynthesis. In soybeans, BR increased photosynthesis and chlorophyll content in soybean leaves during water stress, resulting in enhanced chlorophyll synthesis and photosynthetic enzyme activity (Zhang et al., 2008). In peppers, BR mitigated the adverse effects of drought on photosynthesis by significantly attenuating drought-induced photosynthesis and by increasing the dissipation of excitation energy in leave’s photosystemII (PS II) antennae (Hu et al., 2013). Under the condition of long-term low temperature stress, BR maintained the activity of photosynthetic organs and improved the photosynthetic potential of the tung tree. Along with the maintenance of the stability of the leaf structure, morphology, and function, suffering of tung trees from cold stress could be alleviated (Zhang et al., 2019).

In our study, photosynthetic genes were found to be intensively involved in the BR-accumulation pattern in new shoots of the tea plant, by the KEGG analysis and hub-gene screening. Throughout the forty genes with the top K.in values in both positive and negative modules, up to 42.5% of the genes (with nine genes in positive modules and eight genes in negative modules) were related to photosynthesis, including PLP2, chlorophyll a-b binding protein, photosystem I reaction center subunit XI, ribosomal protein, and so on (Table 1). Most photosynthetic genes originated in chloroplasts, where chloroplasts provide the main place for photosynthesis. It is not hard to deduce that photosynthetic genes played an active role in the BR-accumulation pattern in the new shoots of tea plants. Meanwhile, 90–95% of the biological output directly comes from the products of photosynthesis or at least be affected by photosynthesis, such as sugar, caffeine, tea polyphenols, and other substances, which are the main influencing factors in affecting the quality of tea.

### A Structural Gene Family, CYP450, May Have Core Effect in BR-Accumulation Pattern

The biosynthetic pathway of BR had been clearly studied, including CN-dependent and CN-independent pathways. In the two branches of the biosynthetic pathway, a variety of cytochrome p450 (CYP450) family genes were involved (Figure 1A), and they are homologous. Of all the eight steps in the direct biosynthesis of BL (CN-independent pathways), five genes belonged to the CYP450 family, and in the CN-dependent pathway, CYP450 family genes participate in half of the catalytic process. CYP450, a mono-oxygenase encoded by the supergene family, is a huge family in the plant kingdom and plays an indispensable role in the metabolic reaction of plants (Nebert et al., 1989). The CYP450 family is involved in a variety of secondary metabolic reactions in plants, including the biosynthesis and metabolism of terpenoids, alkaloids, sterols, fatty acids, and phytohormones (Schuler and Werck-Reichhart, 2003).

Precisely, our results showed that CYP450 family genes were intensively screened by the analysis of combining the BR biosynthetic pathway an the K.in value based on WGCNA and expression levels (Table 2, Supplementary Figure S2). Thus, it could conclude that CYP450 family genes may have a core effect in the accumulation of BR and BR-accumulation pattern. It had been implicated in many studies; in rice, *CYP734As* acted as a multifunctional multisubstrate enzyme that controlled the content of endogenous bioactive BR by directly inactivating CS and inhibiting CS biosynthesis by reducing the level of BR precursors (Sakamoto et al., 2011). In pea, two *CYP85As* with CS biosynthetic activity, *CYP85A1* (*Pisum sativum* BR C-6 oxidase 1, also *PsBR6ox1*) and *CYP85A6* (*Pisum sativum* BR C-6 oxidase 6, also *PsBR6ox6*), were capable of converting 6-deoxyribonucleic acid to CS (Jager et al., 2007). These results confirmed our deduction that CYP450 genes had an important role in the accumulation of BR and BR-accumulation pattern. Although there have been many reports showing the important role of CYP450 as a “universal biocatalyst”, the study in other plant species such as the tea tree plant is relatively lagging behind than in the model plant *Arabidopsis thaliana* (Zhu et al., 2019), which needs to be explored in future research.

### TFs Affecting BR-Accumulation Pattern by Regulating Structural Genes in BR Biosynthetic Pathway

**FIGURE 6:**
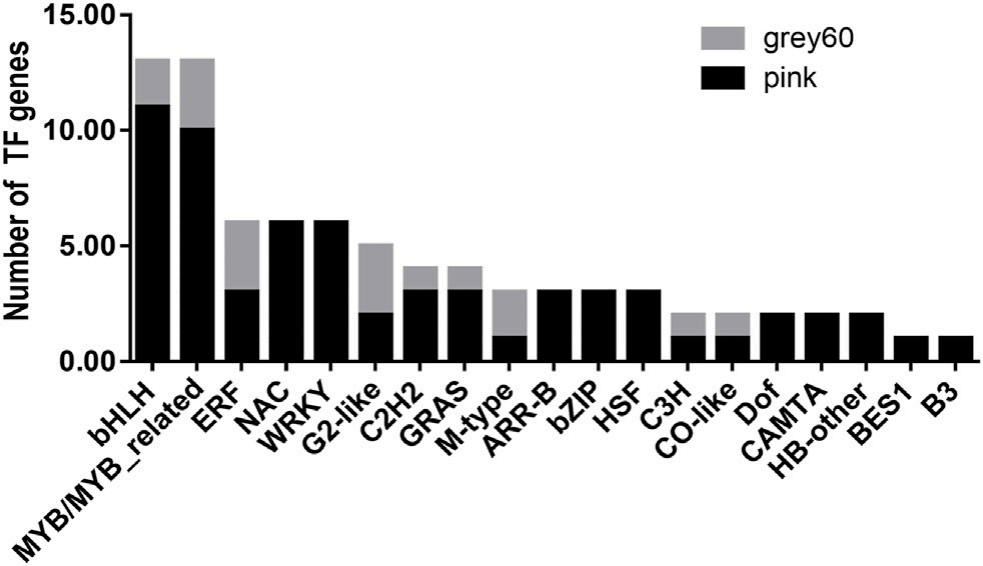
The distribution of TFs in the positive correlation modules and the construction of co-expression network. Types and numbers of transcription factors (TFs) genes in “grey60” and “pink” modules.

As is known, TFs are important components in the transcriptional regulatory process which play an important role in plant growth and development (Lai et al., 2019; Romani and Moreno, 2021). In our study, two kinds of TFs were abundantly enriched in positively correlated modules in the BR biosynthetic pathway, bHLH, and MYB/MYB-related families (Figure 6). Up to now, only a small number of studies are on the TFs affecting the accumulation pattern of BR biosynthesis by regulating BR structural genes.*GhFP1*, a bHLH-coding protein, could activate BR biosynthesis in cotton. Three BR biosynthetic genes, *GhCPD* (*GhCYP90A1*), *GhDWF4* (*GhCYP90B1*), and *GhDET2* were upregulated in *GhFP1* overexpressed fibers and downregulated in *GhFP1* RNAi fibers, respectively (Liu et al., 2020). Besides, a BR biosynthetic gene, *DWF4* (*CYP90B1*) could be regulated by three MYB factors (MYB28, MYB34, and MYB122) in *Arabidopsis* (Guo et al., 2013). Therefore, it is highly likely that TFs such as bHLH and MYB could regulate structural genes in influencing the content and distribution of BR in the new shoots of tea plants.

Another noteworthy thing, a very important TF BRI1-ems-suppressor 1 (BES1) appeared in the “pink” module (Figure 6). According to previous reports, two transcription factors have been identified in the BR signal transduction pathway (Wang et al., 2002; Yin et al., 2002), and BES1 is one of them. After the signal cascade of BR first receptor BRI1 and its co-receptor brassinosteroid insensitive 1(BRI1)–associated kinase receptor 1 (BAK1) was triggered, the negative regulator BRI1 kinase inhibitor 1 (BKI1) was released and the negative regulator brassinosteroid insensitive 2 (BIN2) ceased its activity. This led to the induction and accumulation of two transcription factors BES1 and brassinazole-resistant 1 (BZR1) in the nucleus. Then, activated BES1 and its homologue BZR1 were involved in BR biosynthesis, regulating responsive genes, and affecting plant growth and stress resistance processes (Chen et al., 2019; Li et al., 2019; Cao et al., 2020). BES1 might have been playing an important positive role in the BR signal transduction in the new shoots of tea plants.

### Three Conserved miRNA Families May Play Important Roles in Coordinating the BR-Accumulation Pattern

**FIGURE 7:**
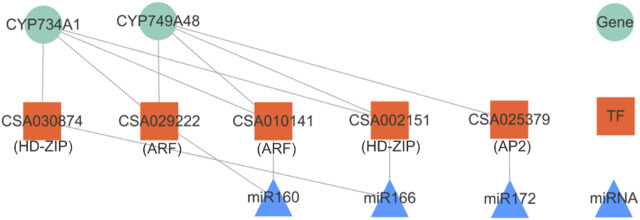
miRNA–TF-target genes network in negative correlation modules. The upper triangle represents miRNAs, the middle square represents TFs, and the lowest circle represents target genes.

MicroRNAs (miRNAs) were first identified in nematodes almost three decades ago (Lee et al., 1993). Since then, plenty of miRNAs genes were identified in animals, plants, and viruses. MiRNAs are post-transcriptional regulators of a large number of target genes by guiding target mRNAs for degradation or by repressing translation (Zhao and Srivastava, 2007). We knew from the above series of results that the BR biosynthetic pathway was more obviously negatively regulated (Table 1, Table 2). In this study, we constructed a miRNA–TF-target gene network in the negative correlation modules. We introduced one-to-one correspondence data of miRNA-TF-target genes from our previous study focused on the conserved miRNAs and then got the regulation network as in Figure 7 (Zhao et al., 2019), including three miRNAs, five TFs and two down-stream target mRNA genes. From the network, miR160, miR166, and miR172 regulate two target genes belonging to the CYP450 family (*CYP734A1* and *CYP749A48*) through three types of TF genes, *ARF*, *HD-ZIP,* and *AP2*. The CYP450 family genes again showed a high correlation in the BR biosynthetic pathway.

Consequently, we could conclude that the conserved miRNAs, miR160, miR166, and miR172, might be indispensable in the accumulation of BR and/or its mechanism. Moreover, another network pair miR5168–*HD ZIP* (*CSA015090*)–*CYP734A51* had also been figured out. Our point of view could also be supported in some studies. In *Arabidopsis*, miR160 promoted hypocotyl elongation through cleavage of its targets *ARF10*, *ARF16*, and *ARF17* in the presence of BRZ (a kind of BR biosynthetic inhibitor) (Dai et al., 2020). In addition, we found a potential regulatory relationship between miR160 and *CYP734A1*, which has not been reported so far. In *Arabidopsis*, miR172 could regulate vegetative growth patterns by modulating BR sensitivity. Overexpression of miR172 could increase its sensitivity to BR, leading to a partial suppression of the leaf phenotypes of *bak1* (Kim et al., 2014).

### Feedback Inhibition Might Dominate the Accumulation Pattern of BR

The above results and analysis, whether based on the K.in value or pathway (ko00905), showed that the negative modules played a dominant role in BR accumulation (Table 1, Table 2). This may suggest that the feedback inhibition of BR is very important for maintaining its content balance in plants (Chung et al., 2012). In *Arabidopsis*, BL treatment reduced the expressions of *CPD/CYP90A1*, *DWF4/CYP90B1*, *CYP90C1*, *ROT3/CYP90D1*, *CYP85A1*, and *CYP85A2* to approximately 10% or less of the level detected in the control group. They found that in *Arabidopsis* all CYP85 and CYP90 gene activities are controlled by BR-dependent feedback regulation (Bancos et al., 2002). In rice, high levels of exogenous BR could induce inactivation of GA and inhibit its biosynthetic pathway, resulting in the decrease of the GA level and ultimately inhibiting the cell elongation. The situation of low levels of exogenous BR is the opposite. On the other hand, high levels of GA could promote the signal transduction of BR, while low levels of GA could inhibit the signal transduction and biosynthetic pathway of BR (Tong et al., 2014).

Many examples of spraying exogenous BR had confirmed that the concentration of BR determines whether it plays a positive or negative regulatory role on growth and development. Within a certain concentration range, spraying exogenous BR could promote tobacco leaf elongation, cell division, and expansion along with the increase in the concentration of BR, thus promoting the growth and expansion of tobacco leaves (Zhang J. et al., 2021). High concentrations of BR negatively regulated the stomata movement by inhibiting ABA-induced stomatal closure and the induction and subsequent production of ROS by ABA on some genes involved in reactive oxygen species (ROS) generation in *Arabidopsis* (Ha et al., 2018). The feedback control of the transcripts of genes in the biosynthetic pathway of BR was also confirmed in our research (related research under review). Besides, the miRNA–TF-target mRNA that might play an indispensable regulatory role could be taken seriously in the future study. It is reasonable to believe that feedback inhibition might dominate the accumulation pattern of BR in tea plants.

## Conclusion

Our study centered on the accumulation of BR and its potential mechanism in the new shoots of tea plants. Positive modules and negative modules were classified based on the combination analysis of the content of BR metabolite and the expression levels of all genes according to the RNA-seq of different tissues in the new shoots. Photosynthesis-relevant genes and CYP450 family genes have considerably high probability in affecting the biosythesis of BR and BR-accumulation pattern. Genes with a high K.in value in negative modules have a crucial effect in the accumulation of BR and/or its mechanism. Feedback inhibition might dominate the accumulation pattern of BR in tea plants. Our findings provided a theoretical basis for further study of the regulation of the BR biosynthetic pathway and the possibility of cross-action studies between BR and other hormones in plants.

All the abbreviations in the manuscript could be found in Supplementary Table S4.

## Data Availability

The original raw data of the 15 libraries were submitted to the public database with the NCBI Sequence Read Archive Database under accession PRJNA781111.
