# Supplementary material for: Feedback Inhibition Might Dominate the Accumulation Pattern of BR in the New Shoots of Tea Plants (Camellia sinensis)
Source: Front Genet. 2022 Feb 22;12:809608. doi: 10.3389/fgene.2021.809608 (PMC8902050; doi:10.3389/fgene.2021.809608)
Supplement: Supplementary file 1 [file DataSheet1.docx]

Supplementary Material

# Supplementary Figures and Tables

## Supplementary Figures

**Supplementary Figure 1.** Heat map showing the correlations between biological replicates. The PCC (Pearson correlation coefficient) values are quantitative indicators of relative expression levels of all genes in each sample.


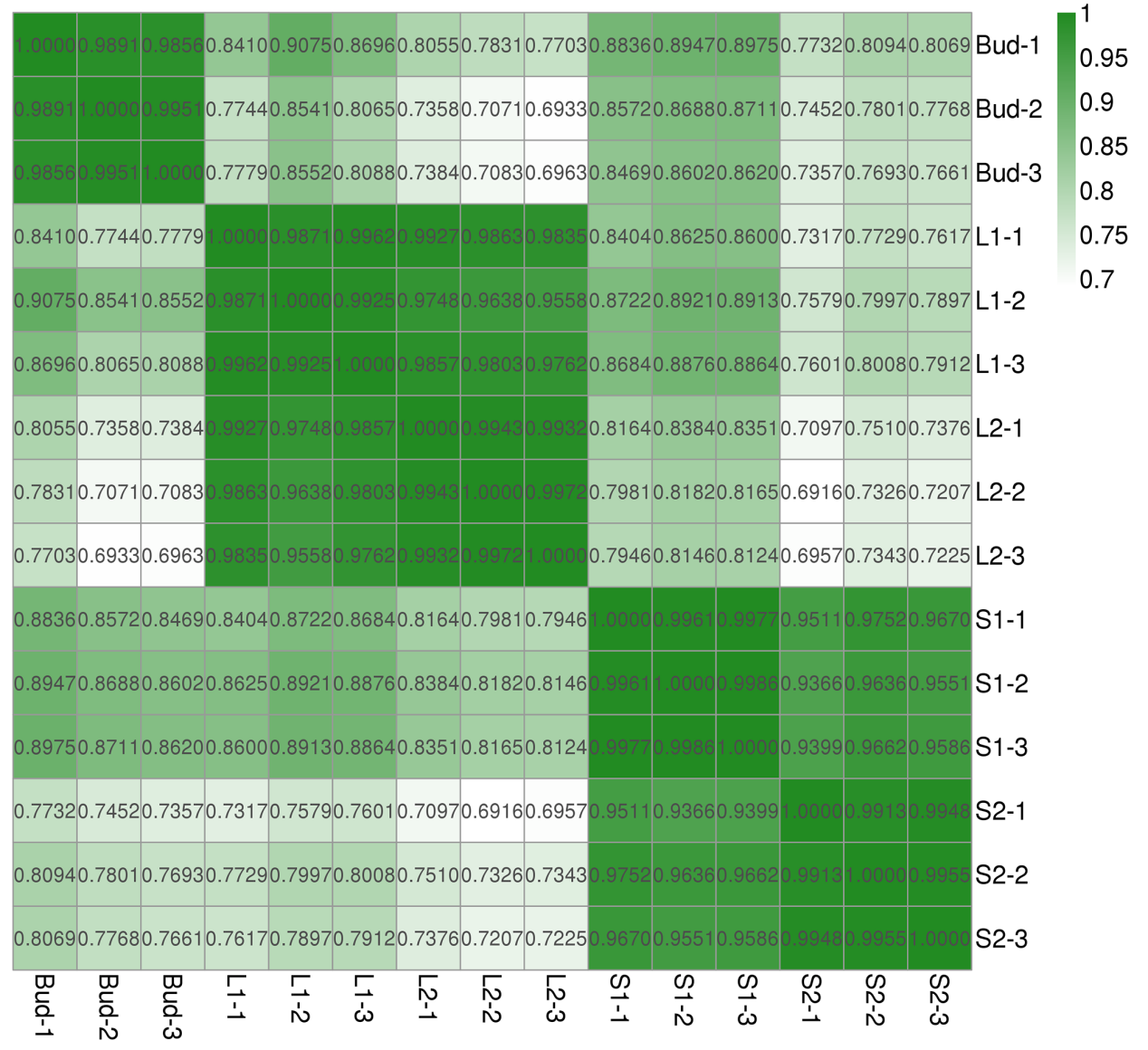


**Supplementary Figure 2.** Relative expressions of the genes related to BR biosynthetic pathway (ko00905) by RNA-Seq in Fragments Per Kilobase of exon model per Million mapped fragments（FPKM）units. Nodes on axis X for each inset image from left to the right are: Bud, L1, L2, S1, and S2. Data values are the means ± SD of three independent biological samples. Lines labeled with different low case letters mean significant difference (p-value less than 0.05), while with the same ones mean insignificance (p-value higher than 0.05).


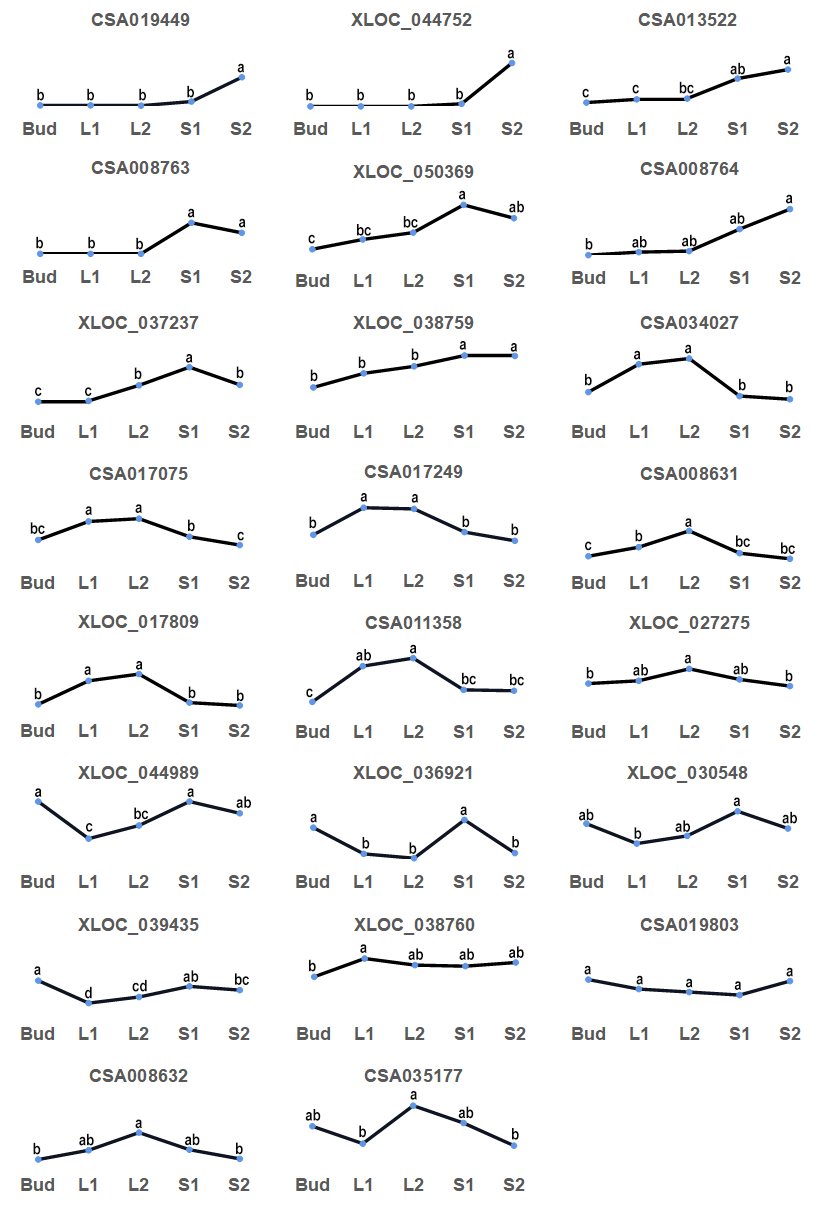


## Supplementary Tables

**Supplementary Table 1.** The content of the five main components of BR in each tissue of tea new shoots.Values: means±SD; (unit: ng•g^-1^ F.W.); n.d.: not detected.

| **Analyte** | **28-norBL** | **BL** | **28-norCS** | **28-homoBL** | **CS** |
| --- | --- | --- | --- | --- | --- |
| **Bud** | n.d. | n.d. | 0.01±0.00 | n.d. | 1.63±0.04 |
| **L1** | n.d. | n.d. | 0.01±0.00 | n.d. | 1.71±0.04 |
| **L2** | n.d. | n.d. | 0.01±0.00 | n.d. | 2.54±0.04 |
| **S1** | n.d. | n.d. | 0.01±0.00 | n.d. | 1.50±0.04 |
| **S2** | n.d. | n.d. | n.d. | n.d. | 0.97±0.04 |

**Supplementary Table 2.** RNA sequencing data from Pingyang tezaotea (PYTZ) new shoots.

| **Sample name** | **Clean reads** | **Q20(%)** | **Q30(%)** | **N(%)** | **GC(%)** | **Mapping Ratio(%)** |
| --- | --- | --- | --- | --- | --- | --- |
|  |  |  |  |  |  |  |
| **Bud-1** | 45898954 | 6512743273 (97.88%) | 6185582044 (92.96%) | 40911 (0.00%) | 3163383682 (47.54%) | 73.18% |
| **Bud-2** | 50967578 | 7347913089 (98.38%) | 7054078317 (94.44%) | 49265 (0.00%) | 3558832714 (47.65%) | 73.57% |
| **Bud-3** | 52449290 | 7566927534 (98.38%) | 7266882027 (94.48%) | 50643 (0.00%) | 3617253783 (47.03%) | 73.45% |
| **L1-1** | 48496694 | 6999286780 (98.46%) | 6732516203 (94.71%) | 47645 (0.00%) | 3367477384 (47.37%) | 71.97% |
| **L1-2** | 47712810 | 6874202893 (98.38%) | 6601346876 (94.47%) | 45633 (0.00%) | 3323638165 (47.56%) | 73.15% |
| **L1-3** | 51174882 | 7361784500 (98.27%) | 7053684973 (94.16%) | 48496 (0.00%) | 3561434846 (47.54%) | 72.46% |
| **L2-1** | 52374348 | 7509086069 (98.32%) | 7204066744 (94.33%) | 49193 (0.00%) | 3634194885 (47.59%) | 72.62% |
| **L2-2** | 53485374 | 7665623378 (98.46%) | 7374605269 (94.72%) | 50801 (0.00%) | 3738225520 (48.01%) | 73.31% |
| **L2-3** | 75426300 | 10681146840 (97.86%) | 10145938641 (92.96%) | 66055 (0.00%) | 5211498098 (47.75%) | 72.08% |
| **S1-1** | 54488592 | 7803142124 (98.41%) | 7500475915 (94.59%) | 51400 (0.00%) | 3798848727 (47.91%) | 71.94% |
| **S1-2** | 70563066 | 10137158616 (98.47%) | 9755770936 (94.76%) | 68221 (0.00%) | 4899322010 (47.59%) | 72.19% |
| **S1-3** | 69092480 | 9925915021 (98.53%) | 9567023474 (94.97%) | 66833 (0.00%) | 4803543038 (47.68%) | 72.47% |
| **S2-1** | 54957944 | 7886770190 (98.44%) | 7566147174 (94.44%) | 60698 (0.00%) | 3863291733 (48.22%) | 72.49% |
| **S2-2** | 57091550 | 8153533201 (98.42%) | 7819419354 (94.38%) | 61500 (0.00%) | 4009594343 (48.40%) | 71.71% |
| **S2-3** | 60699246 | 8691709479 (98.44%) | 8336852793 (94.42%) | 65126 (0.00%) | 4306363292 (48.77%) | 72.35% |

**Supplementary Table 3.** The primers sequences of selected genes used in quantitative real-time polymerase chain reaction (qRT-PCR).

| **Gene ID** | **Primer Sequence(5'-3')** |
| --- | --- |
| CSA011542-Q-F | CCATTGGAAGAGGTGGGTG |
| CSA011542-Q-R | ATTTCCATCGGCAGCATTC |
| CSA002865-Q-F | CGATTATTTGCGAGGCTGCT |
| CSA002865-Q-R | GACCTTCTTCATCCGAGTCTTGA |
| CSA032812-Q-F | CAATCTACCACCATACCAACACCA |
| CSA032812-Q-R | TCCTTCACAGAGTGGGGCTTC |
| CSA005377-Q-F | AGACCTTGGGGAGATGGACG |
| CSA005377-Q-R | TTGGGGCAGCGAAATGAG |
| CSA008632-Q-F | TGTAGGGGTGATGAGAAAGGCA |
| CSA008632-Q-R | CCAAGTCAAGCCCAGGACATAG |
| CSA035177-Q-F | CTCTTGCCCAAACTTGATGACTT |
| CSA035177-Q-R | AGGGTTCCCAACACCAGCTT |
| CSA019338-Q-F | TTCAAGTGCTTCTCCAGTTTCAG |
| CSA019338-Q-R | GCTTTGCCTCTGTTCTTGTTATCT |
| CSA010064-Q-F | AGACCCAAGAAGAGGAGAAGTGA |
| CSA010064-Q-R | ATGGTGTCCAAGGCGGCTA |
| CSA027395-Q-F | GATGTAGTTTGGATGCCCCTGG |
| CSA027395-Q-R | CGGAATGAACAGCACCTAAACG |
| XLOC_044989-Q-F | TCCCAGCAATGACAGCAAGTG |
| XLOC_044989-Q-R | CGATGCCAGGAAAGCGAAT |
| XLOC_030548-Q-F | TTCAAGATTCGCTTTCCTGGC |
| XLOC_030548-Q-R | CACTCCCGTAGTTGTCCGATTTT |
| XLOC_039435-Q-F | TTCAAGATTCGCTTTCCTGGC |
| XLOC_039435-Q-R | CACTCCCGTAGTTGTCCGATTTT |
| CsGAPDH-Q-F | TTGGCATCGTTGAGGGTCT |
| CsGAPDH-Q-R | CAGTGGGAACACGGAAAGC |

**Supplementary Table 4.** List of abbreviations.

| **Abbreviations** | **Full English Name** |
| --- | --- |
| BR | brassinosteroid |
| CS | castasterone |
| CR | campesterol |
| CN | campestanol |
| 6-deoxoCT | 6-deoxocathasterone |
| 6-deoxoTE | 6-deoxoteasterone |
| 6-deoxo-3-DT | 3-dehydro-6-deoxoteasterone |
| 6-deoxoTY | 6-deoxotyphasterol |
| 22-OHCR | (22S)-22-hydroxycampesterol |
| 22-OH-4-en-3-one | (22S,24R)-22-hydroxyergost-4-en-3-one |
| 22-OH-3-one | (22S,24R)-22-hydroxy-5-ergostan-3-one |
| 6-deoxoCS | 6-deoxocastasterone |
| 28-norBL | 28-norbrassinolide |
| 28-homoBL | 28-homobrassinolide |
| 28-norCS | 28-norcastasterone |
| TFs | Transcription Factors |
| UPLC-MS/MS | Ultra Performance Liquid Chromatography Tandem Mass Spectrometry |
| PYTZ | Pingyang Tezaocha |
| KEGG | Kyoto Encyclopedia of Genes and Genomes |
| FPKM | Fragments Per Kilobase of transcript per Million mapped reads |
| WGCNA | Weighted Gene Co-expression Network Analysis |
| MM | module connectivity and module correlation |
| TOM | Topological Overlap Matrix |
| qRT-PCR | Quantitative Real-time Polymerase Chain Reaction |
| BAH | Bromo adjacent homology |
| PS II | Photosystem II |
| CYP450 | Cytochrome p450 (CYP450) |
| BES1 | BRI1-ems-suppressor 1 |
| BRI1 | Brassinosteroid Insensitive 1 |
| BIN2 | Brassinosteroid Insensitive 2 |
| BZR1 | Brassinazole-resistant1 |
| BAK1 | Brassinosteroid Insensitive 1（BRI1）Associated Kinase Receptor 1 |
| miRNAs | MicroRNAs |
| PHB | Phabulosa |
| PHV | Phavoluta |
| REV | Revoluta |
| ATHB | Arabidopsis Thaltana Homeobox |
| ROS | Reactive Oxygen Species |
| FPKM | Fragments Per Kilobase of transcript per Million mapped reads |
